# Muscle fascicle length adaptations to high-velocity training in young adults with cerebral palsy

**DOI:** 10.3389/fspor.2025.1558784

**Published:** 2025-05-13

**Authors:** Tessa L. Gallinger, Brian R. MacIntosh, Jared R. Fletcher

**Affiliations:** ^1^ Human Performance Laboratory, Faculty of Kinesiology, University of Calgary, Calgary, AB, Canada; ^2^ Canadian Paralympic Committee, Ottawa, ON, Canada; ^3^ Department of Health and Physical Education, Mount Royal University, Calgary, AB, Canada

**Keywords:** sarcomerogenesis, neurological disorder, power, sprint agility, force-length, force-velocity, para sport

## Abstract

**Introduction:**

In individuals with Cerebral Palsy (CP), both muscle cross-sectional area and fascicle length are reduced, contributing to decreased muscle strength, muscle shortening velocity and muscle mechanical power output, particularly in the plantarflexor muscles. A proposed mechanism to target increased muscle mechanical power output is to incorporate high velocity training (HVT) in these individuals, to increase fascicle length via sarcomerogenesis. To determine the effects of HVT on changes in MG muscle fascicle length and that impact on changes to MG muscle force-length-velocity-power characteristics in young adults with CP.

**Methods:**

12 young adults with CP (GMFCS I or II, 22.8 ± 6.0 years) were randomly allocated (some crossover) to no training (CP-NT, *n* = 8), or training (CP-T, *n* = 8). 10 recreationally trained healthy adults (HA, 22.5 ± 2.8 years) served as controls. CP-T performed 10-week training of biweekly sessions consisting of progressive intensity 10 m sprints, plyometrics and agility tasks. Triceps surae muscle force-power-velocity relationships were quantified with isokinetic dynamometry and ultrasound imaging. Data are expressed relative to pre-intervention values.

**Results:**

HVT resulted in a significant increase in fascicle length in CP-T (+1.92 ± 3.21 mm, *p* < 0.005) compared to a significant decrease in CP-NT (−1.63 ± 3.00 mm, *p* < 0.013). While HVT did not result in significant changes in maximal shortening velocity (V_max_) or maximal peak power output (P_max_), a large effect size for v_max_ following training in CP-T was seen (+45.2 ± 76.4%, *d* = 0.909, *p* = 0.452), in contrast to CP-NT (+2.9 ± 70.5%, *d* = 0.059, *p* = 1.00). HVT also resulted in a very large effect for P_max_ in CP-T (+35.0 ± 49.1%, *d* = 1.093, *p* = 0.232), but only a small effect was observed in CP-NT (+7.8 ± 49.1%, *d* = 0.245, *p* = 1.00). HA had significantly greater P_max_ (*p* < 0.001), longer resting and active fascicle lengths (*p* < 0.001) and greater muscle force (*p* < 0.001), compared to CP-T.

**Discussion:**

HVT is a feasible training intervention to increase triceps surae muscle fascicle length in individuals with CP. HVT can partially mitigate losses in P_max_ in CP compared to healthy adults. Longer HVT programs may be required to increase muscle mechanical power output in CP to levels observed in HA.

## 1 Introduction

Successful sport performance is largely dependent on production of muscular power, or an ability to achieve maximal force generation at high muscle shortening velocities. Muscular power is influenced by specific muscle-tendon properties that can be optimized through targeted training interventions. For individuals with cerebral palsy (CP) to compete at a high level in sport, it is critical to train in ways that enhance muscle architectural properties, leading to functional improvements. Due to an upper motor neuron lesion, individuals with CP present with a series of permanent movement disorders and secondary adaptations to muscle structure, function and composition^1^. This includes impaired muscle growth as early as 12 months after birth, impacting overall muscle volume (1). A reduced muscle volume may result from shorter muscle fascicle lengths and/or smaller physiological cross-sectional area (PCSA) (2), reducing the number of sarcomeres working in series and/or in parallel. While a muscle's PCSA is directly related to its force output, muscle length is an important determinant of a muscle's maximal shortening velocity (3). In CP, shorter muscle fascicle lengths have been found, compared to those of healthy adults (HA) without a neurological disorder (4, 5), where it appears that serial sarcomere number is reduced and/or longer sarcomere lengths are also present (6–8). These differences in muscle structure can contribute to muscle weakness, limited range of motion (ROM) and increased passive stiffness in even high functioning individuals with CP (9).

The muscle weakness observed in CP is typically more prevalent and pronounced in the distal muscle groups (10), adversely affecting ambulation. Specifically, reduced preferred and fast walking speeds, which have been correlated with decreased muscle strength, rate of force development and mechanical power generation at the ankle joint in CP (11–14). Increasing plantarflexor power may contribute to a greater velocity of the centre of mass at the end of the propulsive phase of running and jumping, as well as improving ambulation in CP (13, 14). A proposed mechanism to target improvements in muscular power, is to incorporate strength training with relatively high velocity contractions, with the intent to increase the number of sarcomeres in series (15). This process is referred to as sarcomerogenesis, where an increase in number of sarcomeres within a muscle fascicle, can generate a higher relative force for a given muscle shortening velocity (13, 16). Although it is now well understood that maximal effort and high-intensity exercise can be implemented safely, and without adverse effect on muscle spasticity (14, 17–20), studies incorporating high-velocity training (HVT) are limited. To date, only two studies have evaluated changes in fascicle length with HVT for people with high-functioning CP (Gross Motor Classification, GMFCS I-III) (13, 21). Moreau et al. (2013) observed increases in rectus femoris fascicle length, but not in the vastus lateralis muscle, following progressive isokinetic training at angular velocities of 30–120°•s^−1^. These slow movement velocities cannot be considered within our definition of true HVT (or sport-specific training velocity) as a comparison, individuals without a neurological disorder can reach knee extension velocities of 800°•s^−1^ during the push-off phase of a jump (22), and approximately 600°•s^−1^ during mid-stance sprint running (23, 24). Alternatively, Gillet et al. (2018) did include maximal short-distance sprint training (HVT), but this study reported no changes to medial gastrocnemius fascicle length following the intervention (21). Both studies did, however, observe increases in maximal strength, muscle power and total muscle volume, indicating a positive effect of resistance training on muscle cross-sectional hypertrophy in CP.

There appears to be potential to improve functional mobility of individuals with CP, as well as potential to change muscle architecture with training. However, the literature on sarcomerogenesis and/or use of HVT in CP is still limited, constraining our ability to prescribe specific and targeted training protocols. Additionally, a majority of studies have concentrated their assessments of function on joint moment data in CP, leaving gaps in our understanding of the specific skeletal muscle mechanical properties adaptations from exercise. Understanding how changes in fascicle length can alter the muscle's force-length and force-power-velocity relationships is critical to our understanding of how muscle mechanics influence movement and performance in individuals with CP. If mechanical power is to be maximized, then muscles should operate on the plateau of the force-length and the power-velocity curves (25). This knowledge could inform targeted training interventions in this population to optimize muscle function and improve muscle power output. In clinical situations, information about force-length and force-velocity properties could be used to determine the amount of muscle or tendon elongation is required to improve plantarflexion function during ambulation (26, 27). Therefore, the primary purpose of this study was to determine the effects of HVT on changes in MG muscle fascicle length and that impact on changes to MG muscle force-length-velocity-power characteristics in young adults with CP. We hypothesized that HVT would lead to increases in resting fascicle length and translate to increased triceps surae muscle power output.

## 2 Materials and methods

### 2.1 Participants

12 ambulatory participants with CP and 10 recreationally trained young healthy adults (HA) without a neurological disorder participated in this study. Participant characteristics can be found in Table 1. All participants were injury-free and had no lower limb surgery or botulinum neurotoxin A (Botox) injections within 6 months prior to the testing. Individuals training 3 or more times per week were defined as recreationally active (*n* = 8), and those who were identified as sedentary (*n* = 4) were not meeting Canada's Physical Activity Guidelines within the last 6 months. HA participants included those who were participating in sprint-agility training 3 or more times per week.

**Table 1:** Participant characteristics.

Group	*n*	Male/female	Age	Height	Mass	Diplegia/Hemiplegia	GMFCS
		(n)	(years)	(m)	(kg)	(n)	(n)
HA	10	4/6	22.5 ± 2.8	1.67 ± 0.09	67.8 ± 8.8	n.a.	n.a.
CP	12	8/4	22.8 ± 6.0	1.65 ± 0.11	61.9 ± 12.8	6/6	7 I, 5 II

Values are mean ± SD.

Abbreviations: n, number of participants; HA, healthy adults; CP, cerebral palsy; GMFCS, gross motor classification scale; GMFCS I, Ambulatory—no assistance; GMFCS II, Ambulatory—some assistance for long distances;m, meters; kg, kilograms.

### 2.2 Interventions

**Figure 1:**
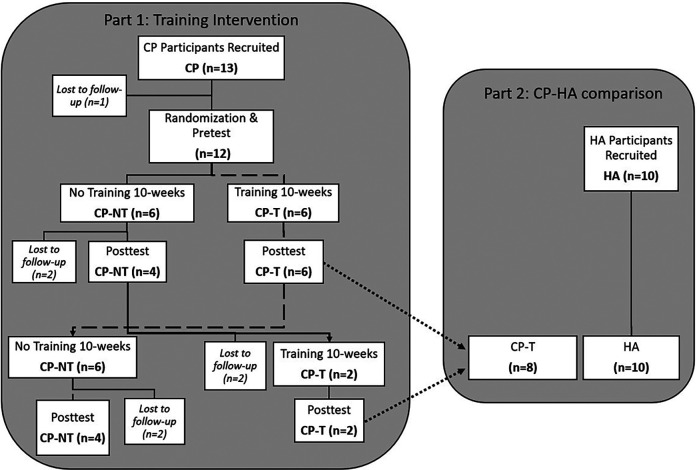
Flowchart depicting a cross-over design for the two-part study. Dashed line indicates the flow for pre and posttest assessments in the training group (CP-T), with a cross-over to the no-training intervention (CP-NT). Dotted line indicates CP-T comparison to HA. CP, cerebral palsy; NT, no training; T, training; HA, healthy adults.

**Figure 2:**
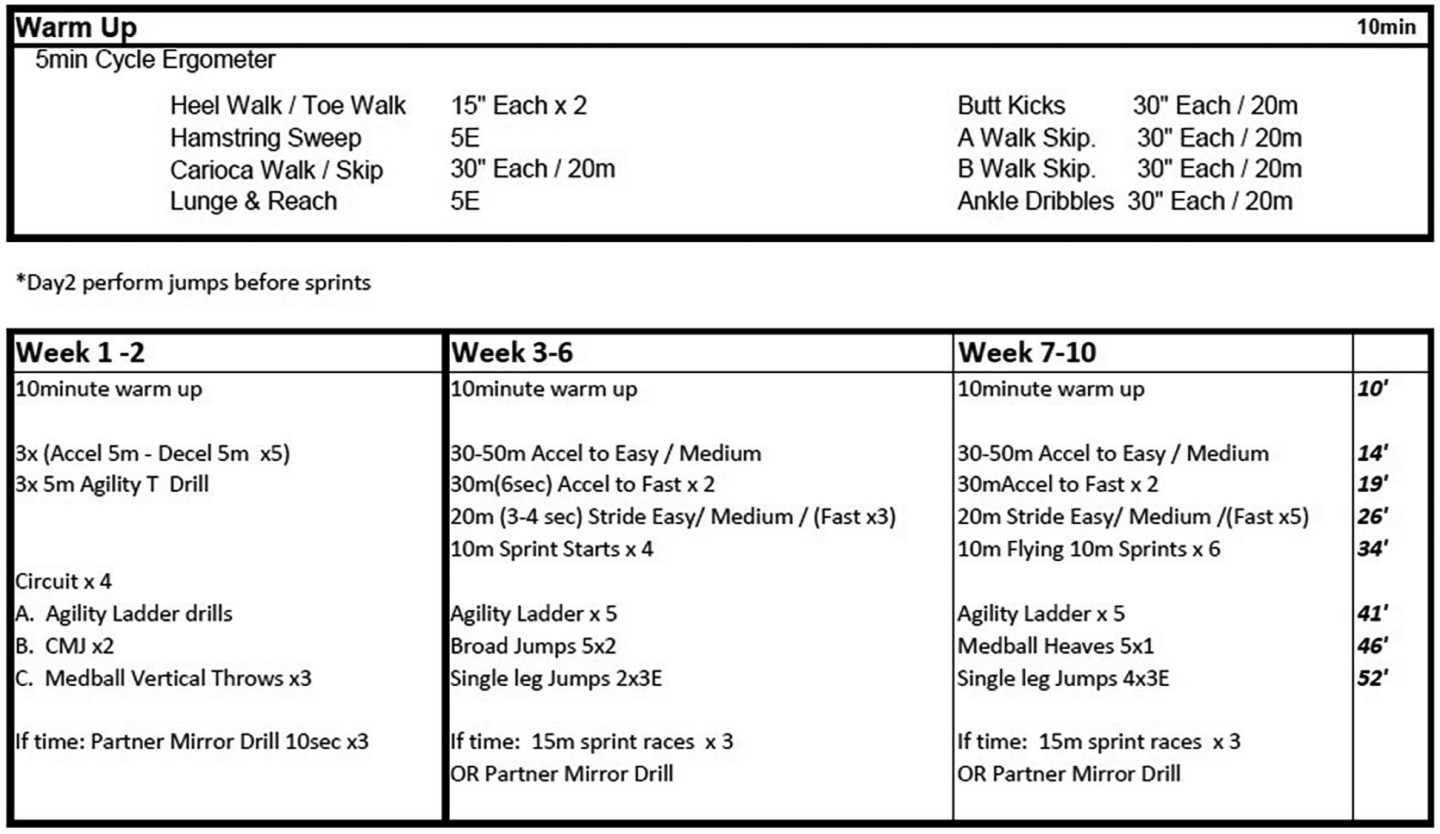
High-velocity training program for CP-T participants weeks 1-10. Multiplication values indicates number of sets completed, or number of repetitions x number of sets. Time allotted to each exercise set is on the right-hand side, displayed in minutes. m, meters; E, each leg; Accel, accelerations; Decel, decelerations; CMJ, countermovement jump;.

To test the hypothesis that relatively high movement velocity, which we term “high velocity training” (HVT) here, would alter muscle architecture and muscle mechanical properties, the CP participants (*n* = 12) were randomly allocated in a cross-over design to either no training (CP-NT) (*n* = 6), or training (CP-T)(*n* = 6) for 10 weeks (Figure 1). The training program performed by the training group can be found in Figure 2. Within the CP-NT group, 2 participants dropped out of the study prior to the post assessment and 2 participants completed the training after serving first as a participant in the no-training (CP-NT) group. Within the CP-T group, 4 participants served as participants in the CP-NT group after completing the training. This resulted in 8 participants comprising each group, and each participant completed a pre and post intervention assessment 10 weeks apart. In cases where a crossover occurred, the first posttest assessment served as the pretest assessment for the subsequent condition. Following completion of interventions, the CP-T group (*n* = 8) and CP-NT group (*n* = 8) were compared to assess the effects of HVT on muscle architecture and muscle mechanics. In part 2 of the study, post-training results for the CP-T group (*n* = 8) were compared to a group of healthy adults (HA) (*n* = 10) who completed one testing assessment. Participants gave their informed written consent to participate in the experimental procedures prior to data collection. The University of Calgary Conjoint Health Research Ethics Board approved the experimental protocol (REB15-2026).

### 2.3 Muscle architecture

**Figure 3:**
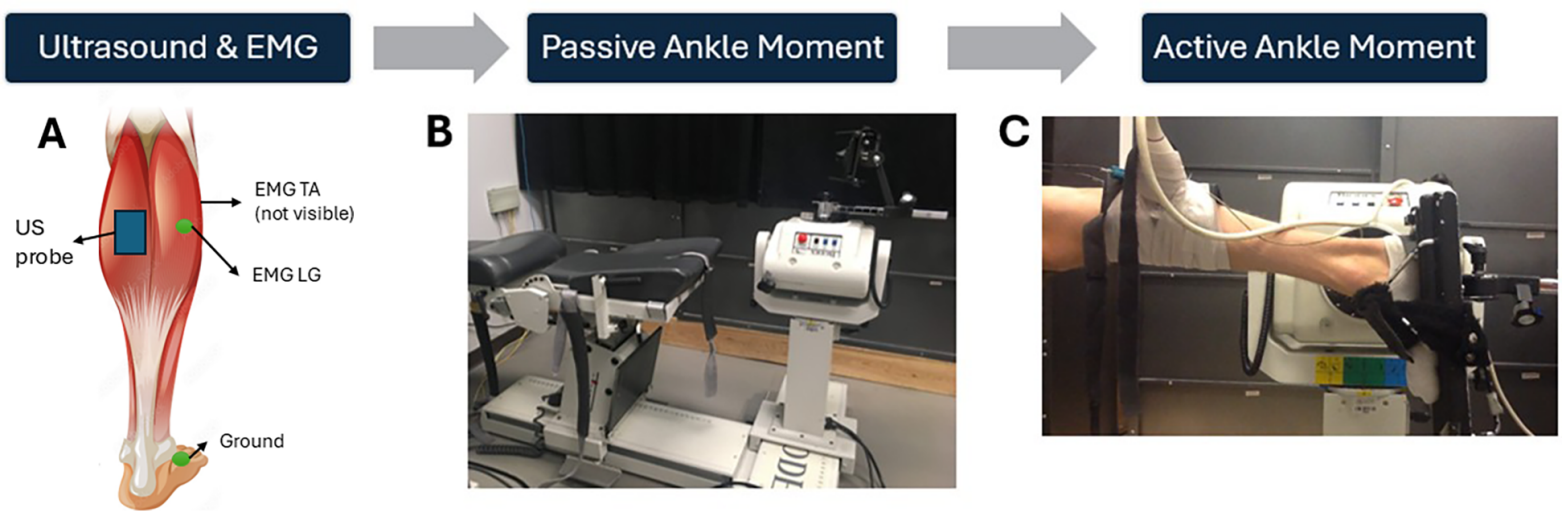
Experimental set-up. Placement of the ultrasound probe on the MG and sEMG electrodes on the LG and TA muscles, respectively **(A)** The measurement of passive moment, avoiding any passive moment due to the weight of the foot and gravity, by having participants side-lying during the passive assessments **(B)** Measurement of active ankle plantar flexor moment with the participant lying prone. **(C)** Adapted with permission from Muscle Stock Vector | Adobe Stock, File # 83745249 by blueringmedia, licensed under standard license, stock.adobe.com.

The experimental set up for the measurement of passive and active moments is shown in Figure 3. Participants were positioned on their side for passive measurements and prone for active measurements with one foot affixed to the dynamometer footplate (Biodex System 3, Shirley NY). Passive measurements were performed in the horizontal plane to remove any gravitational moments resulting from the weight of the footplate and the foot (28). The right foot was used unless the left leg was more affected in any of the CP participants. Images of the deep and superficial aponeuroses and medial gastrocnemius (MG) fascicles were acquired at 49 Hz using Ultrasound (12.5 MHz linear array, Philips Envisor, Eindhoven, Netherlands) imaging to a depth of 3 cm.

Two surface electromyography (EMG) electrodes (Norotrode 20 bipolar Ag-AgCl electrodes, Myotronics Inc, Kent, WA, USA) monitored muscle activation of the tibialis anterior (TA) and lateral gastrocnemius (LG) muscles, with a ground electrode over the medial malleolus. The ultrasound probe prevented monitoring EMG of the MG, so the LG was chosen as a surrogate (29). The EMG window length used to calculate RMS was 100 ms around the peak MVC (i.e., 50 ms prior to 50 ms after peak MVC). Muscle activation was defined as any EMG root mean square (RMS) exceeding three standard deviations (SD) from baseline noise (measured over a 3 s window prior to the trial); however, this criterion was not met in any passive trial. Co-activation of the TA muscle during the isokinetic and isometric MVC trials, was quantified relative to the LG muscle activation (TA:LG). Medial gastrocnemius fascicle length was measured during the passive and active trials using publicly available image analysis software (*Image J*, Baltimore, MD).

### 2.4 Muscle mechanics

#### 2.4.1 Passive moment-fascicle length relationship

In the side lying position, passive rotation of the ankle by the dynamometer occurred through the full range of motion at 0.17 rad·s^−1^ to avoid initiating a stretch reflex (30), as confirmed by passive moment and EMG measurements. Maximal DF (0% relative ankle ROM) and maximal PF (100% relative ankle ROM) were determined with the dynamometer by having the participant actively dorsiflex and plantarflex the ankle. Fascicle length, passive ankle moment and absolute ankle angles were recorded at each participant's fixed relative percentage (0-20-40-60-80-100%) of ankle ROM. Passive moment at any ankle angle could then be estimated using a passive moment-fascicle length quadratic formula. Resting fascicle length at a common ankle angle of 105°, was also compared across CP and HA participants.

#### 2.4.2 Active moment-fascicle length relationships

Isometric MVCs of the plantarflexors were completed by each participant at a fixed relative percentage (0-20-40-60-80-100%) of their ankle ROM 0% was considered full dorsiflexion and 100% was considered full plantarflexion. Participants were provided with a two-minute rest period between repetitions at each joint angle. The order of angle was randomized for each participant. Absolute ankle angles were recorded at each relative ankle angle, and the highest peak active isometric plantarflexion moment of 3 maximal efforts were analyzed. Active plantarflexion moment was calculated by subtracting the passive dorsiflexion moment measured at the corresponding fascicle length, thereby accounting for fascicle length changes (and reduced passive dorsiflexion moments) during contraction (31, 32). MG active fascicle length (the fascicle length associated with peak MVC at each angle), resting fascicle length (mm), pennation angle (deg) and muscle thickness (mm) were recorded at the ankle angle where peak isometric moment occurred. Mean data were obtained by averaging the measured variables across ankle angle (as a %ROM).

Muscle fascicle shortening (as a % of resting fascicle length) was calculated as:

[(resting fascicle length – active fascicle length)/resting fascicle length] · 100

#### 2.4.3 Active moment-active power-angular velocity relationships

Following the isometric trials for the determination of participant-specific force-length relationships, a three-minute break was provided before participants performed isokinetic MVCs at six pre-determined angular velocities (30, 60, 120, 180, 300, 500°•s^−1^) in a randomized order. While the dynamometer was set at these pre-determined angular velocities, not all participants achieved those fixed absolute angular velocities. Therefore, the peak isokinetic angular velocity achieved was recorded for each trial, and fascicle length at peak isokinetic velocity was measured to calculate active moment at that peak measured angular velocity. Participants completed three MVCs consecutively per trial and were provided a two**-**minute rest period between trials at each angular velocity.

Muscle maximal power output (P_max_) and maximal angular velocity (*ω*_max_) can be determined with estimation by fitting moment-angular velocity data to the Hill equation (33), or by linear regression analysis (34). The Hill equation yields a hyperbolic relationship between angular velocity and moment, but lacks precision at high and low velocities. Mid-range of the hyperbola appears to fit moment-angular velocity data well (34). In addition, the angular velocity at which maximal power occurs is termed optimal angular velocity (*ω*_opt_) and has been determined using moment-angular velocity data for both linear regression and fitting to the Hill equation (35, 36). Therefore, a linear regression equation was fit to the active moment-angular velocity data, using the measured peak isometric moment determined from the active moment-fascicle length relationship, as the y-intercept (M_o_). Moment at any angular velocity (*ω*) can then be determined using the calculated slope (m) and y-intercept (M_o_) according to the equation:

$$\mathrm{Moment}\;=\;m\omega \;+\;M_{o}$$

Peak angular velocity (rad•s^−1^) was estimated as the x-intercept of the fitted moment-angular velocity relationship, and can be calculated where moment *=* *0,*

ω=−moment/m

Peak angular velocity and peak moment (M_o_) at peak angular velocity were recorded for all participants. Optimal velocity (ω_opt_) was determined as 50% of the estimated maximal angular velocity. Where, moment = mv + M_o_*,* power can be calculated at any angular velocity as:

$$\mathrm{Power}\;=\;\mathrm{moment}\cdot_{\omega }$$

Peak power (P_max_) was calculated as the moment or force generated at ω_opt_:

$$P_{\max}\;=\;\omega_{\mathrm{opt}}\cdot {0.5M}_{o}$$

This point also corresponds to where slope of the power-angular velocity relationship was equal to zero. Regression values were used instead of observed/measured values for the product of peak moment and the corresponding angular velocity.

#### 2.4.4 Triceps surae moment arm

Triceps surae muscle moment arm (MA) for each participant was quantified using both the tendon travel method (37), and the visual method (38, 39). In a previous study, the visual method resulted in a smaller mean bias (0.8 mm, CI: −1.80 to 2.78 mm) between test/retest compared to the tendon travel method (6.2 mm, CI: −16.0 to 11.3 mm, 153 *p* < 0.001) (40). Therefore, only values from the visual method were used in the current study. Muscle force was then calculated as the ratio of the plantarflexion moment generated during the trial and the estimated triceps surae muscle MA.

Peak muscle force (F_max_) was calculated using the estimated MA and calculated peak moment (M_o_):

$$F_{\max}\;=\;M_{o}/\;MA$$

#### 2.4.5 Muscle force-power-velocity relationship

Peak muscle shortening velocity (V_max_) was calculated during peak isokinetic MVC as the change in fascicle length over contraction time:

ΔFascicle length = resting fascicle length – active fascicle length

Contraction time = Δtime at peak angular velocity ± 15 ms

V_max_ = Δfascicle length/contraction time

Muscle shortening velocity, muscle force, and muscle power were expressed as a percentage of pre-intervention peak across all isokinetic trials and participants to assess the changes in the force-velocity regression relationships following the intervention period. This allowed for a clearer analysis of potential changes between the CP-T and CP-NT groups by reducing the variability observed in the raw data.

### 2.5 Statistics

Statistical analyses were conducted using JASP (Version 0.19.1; JASP Team, 2024). For all analyses a greenhouse-geisser correction was used where mauchlys test indicated that the assumption of sphericity was violated. The Holm *post hoc* analysis was used to test for significant differences. The *a priori* level of statistical significance was considered for p **≤** 0.05. Values are presented as mean ± SD.

#### 2.5.1 Part I – training intervention

An independent *t*-test determined the differences between CP-T and CP-NT groups at baseline for age, height, and weight. To assess changes in the force-length relationship, a two-way repeated measures analysis of variance (ANOVA) was used to test for significant effects of group (CP-T or CP-NT) and time (pre and post-intervention) on ROM and measures at peak isometric MVC (muscle thickness, pennation angle, fascicle shortening percentage, active fascicle length, peak moment, peak force, and EMG RMS TA and LG). A three-way repeated measures ANOVA was used to test significant effects of group (CP-T and CP-NT), time (pre and post intervention), and percentage of ankle ROM (0%, 20%, 40%, 60%, 80%, 100%) on passive moment, resting fascicle length, percentage peak active force, and active fascicle length. Measured peak isometric moment and estimated isometric moment were compared across MVC trials using an independent *t*-test, as well as an assessment of the fit and slope of the moment-angular velocity relationships using the measured and estimated isometric moments. To assess changes in the force-velocity-power relationship, a two-way repeated measures ANOVA was used to test for significant effects of group (CP-T or CP-NT) and time (pre and post-intervention) on mean relative changes to V_max_, V_opt_, F_max_, P_max_ and slope of the force-velocity relationships. A three-way ANOVA was used to test significant effects of group (CP-T and CP-NT), time (pre and post intervention), and isokinetic angular velocity (30, 60, 90, 120, 300, 500°s^−1^) on mean relative muscle shortening velocity, muscle force and power output.

#### 2.5.2 Part II – CP compared to HA

An independent *t*-test determined the differences for age, height, weight, ankle ROM, and moment arm. At peak isometric MVC, a one-way ANOVA compared groups on peak moment, muscle thickness, pennation angle, muscle fascicle shortening percentage, and EMG RMS for the LG and TA. A two-way repeated measures ANOVA was used to test significant effects of group (CP-T and HA) and percentage of ankle ROM (0, 20, 40, 60, 80, 100%) on passive moment, peak force, active fascicle length and resting fascicle length. A two-way repeated measures ANOVA was used to test significant effects of group (CP-T and HA) and isokinetic trial (30, 60, 90, 120, 300, 500°s^−1^) on peak force, peak velocity and peak power.

## 3 Results

### 3.1 Part I - training intervention

#### 3.1.1 Participant characteristics

At baseline there were no significant differences between CP-NT and CP-T for age, height, or weight (Table 1). No significant group (CP-T and CP-NT)×time (pre and post intervention) interaction in maximum dorsiflexion angle, maximum plantarflexion angle, or total ROM was observed. A main effect of time on dorsiflexion angle was found for CP-NT, [*F*(1) = 10.5, *p* = 0.014].

#### 3.1.2 Passive moment-length relationship

**Figure 4:**
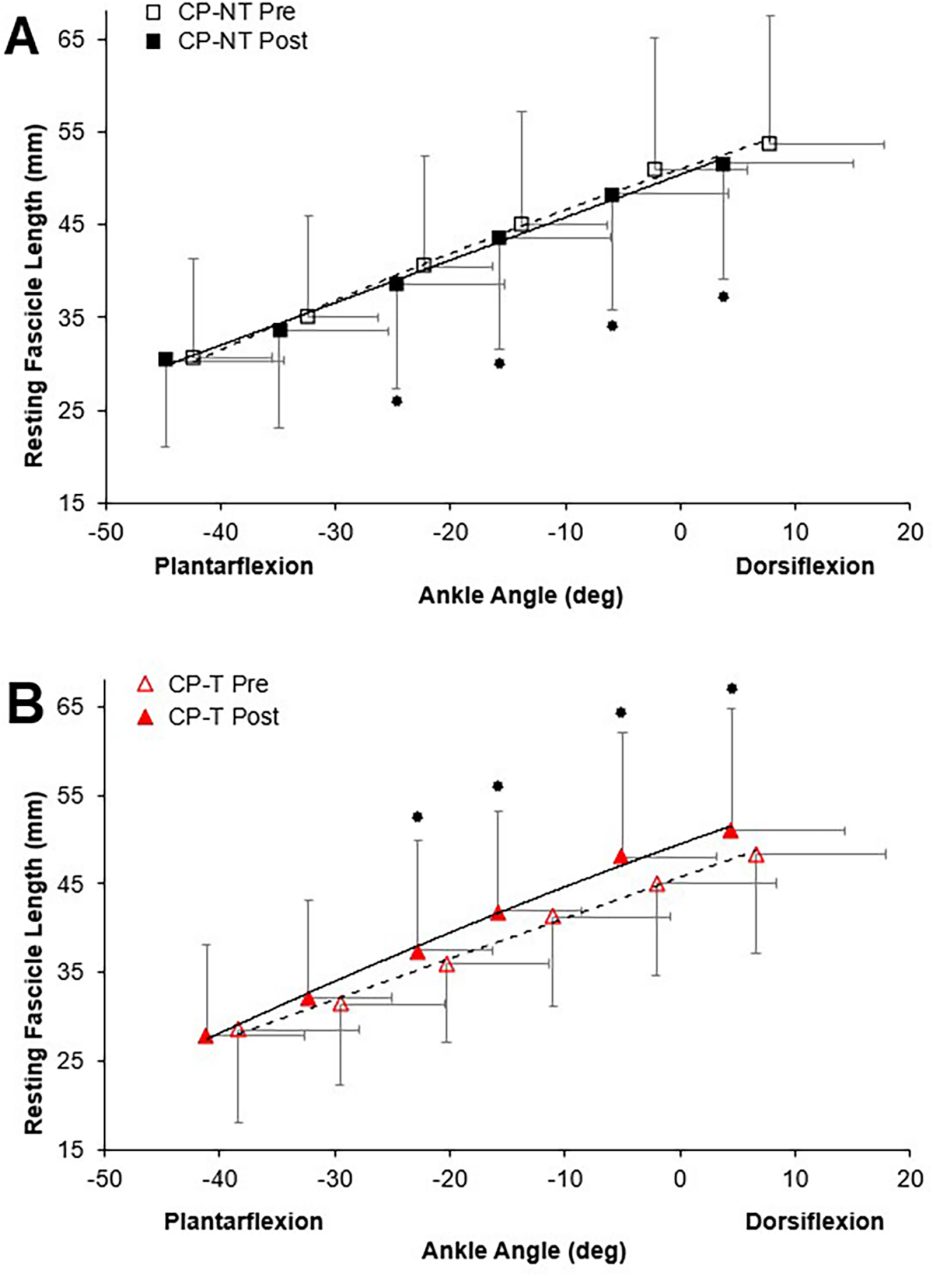
Resting fascicle length-angle relationships in the MG muscle for pre (dashed line, open symbol) and post (straight line, filled symbol) intervention during passive ankle rotation. Actual ankle angles are presented. Data are mean *±* SD. 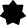 Indicates significant group x time x ankle angle interaction (*p* < 0.04). **(A)** CP-NT (black 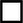, 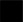). **(B)** CP-T (red 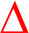, 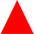).

Passive ankle moment, during the passive rotation trial, did not result in any meaningful muscle activation above the baseline noise, confirming the absence of a stretch reflex and/or any active moments generated during the passive trials. There were no significant three-way group x time x ankle angle interactions for passive moment. There was a significant three-way group x time x ankle angle interaction for resting fascicle length (*p* = 0.038, Figure 4). Training resulted in an increased mean resting fascicle length at 40, 60, 80% and 100% of ROM in CP-T (*p* < 0.005), compared to a significantly shorter resting fascicle length at similar ankle ROM in CP-NT (*p* < 0.013).

#### 3.1.3 Muscle activation

Co-activation of the TA and LG were apparent during the isometric and isokinetic trials for the CP participants. There were no significant two-way group x time interactions for co-activation ratios calculated at peak isometric MVC, or main effects of group or time. EMG RMS for both LG and TA can be found in Table 2, along with the primary outcome measures at MVC for CP-T and CP-NT, pre and post intervention.

**Table 2:** Mean primary outcomes during MVCs for CP-T & CP-NT.

Variable	CP-NT		CP-T		*p*-value
	Pre	Post	Pre	Post	
Muscle thickness (mm)	13.79 ± 2.05	13.90 ± 1.90	13.42 ± 2.3	13.96 ± 2.10	0.895
Pennation angle (deg)	17.59 ± 3.82	18.68 ± 3.83	20.08 ± 4.15	21.06 ± 6.11	0.384
Fascicle shortening (%)	41.04 ± 11.6	46.06 ± 14.13	44.78 ± 16.4	45.63 ± 11.9	0.638
Active fascicle length (mm)	30.80 ± 8.81	31.73 ± 8.71	27.29 ± 8.22	29.25 ± 4.76	0.410
Peak moment (Nm)	59.69 ± 19.6	62.91 ± 22.3	54.84 ± 21.6	55.31 ± 23.8	0.548
Peak force (N)	2,191.1 ± 892.3	2,333.8 ± 981.0	1,849.4 ± 714.5	1,614.2 ± 631.3	0.209
EMG RMS LG	0.17 ± 0.17	0.07 ± 0.05	0.09 ± 0.06	0.11 ± 0.06	0.522
EMG RMS TA	0.07 ± 0.09	0.03 ± 0.01	0.04 ± 0.03	0.04 ± 0.01	0.594

Values are mean ± SD*.*

Abbreviations: CP-NT, cerebral palsy-no training group; CP-T, cerebral palsy-training group; EMG RMS, electromyography root mean square; LG, lateral gastrocnemius muscle; TA, tibialis anterior muscle; mm, millimeters; deg, degrees; %, percentage; Nm, Newton meter; N, Newton.

#### 3.1.4 Force-length relationship

**Figure 5:**
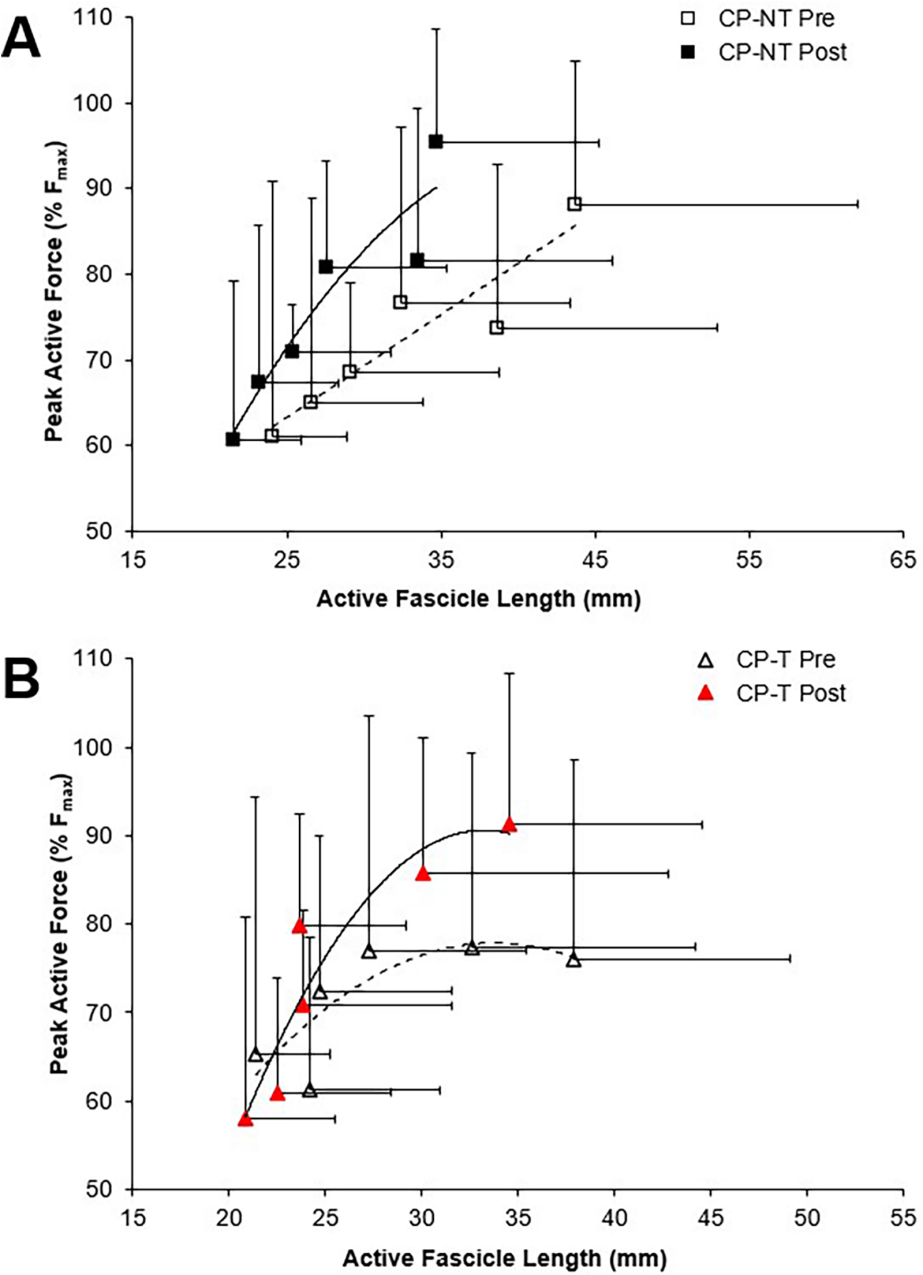
Force-length relationships for the MG muscle during pre (dashed line, open symbol) and post (straight line, filled symbol) intervention during isometric MVCs. Data are mean ± SD. Isometric force is presented as the percentage of peak active force. **(A)** CP-NT (black 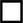, 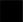) **(B)** CP-T (red 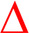, 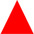).

The muscle force-fascicle length relationship prior to and following the intervention period is shown in Figure 5. During the isometric MVCs, there were no significant three way group x time x ankle angle interactions for active fascicle length, or changes in percent peak force. A significant group x time interaction was demonstrated, indicating a significantly shorter average active fascicle length following the intervention period in CP-NT (*p* < 0.05), while remaining similar following the intervention period in CP-T (*p* = 0.814).

#### 3.1.5 Moment-power-angular velocity relationship

Using a linear regression to fit the measured moment-angular velocity data, the measured peak isometric moment was significantly higher than the estimated maximal isometric moment (mean difference = 22.4 ± 8.6 Nm, *p* < 0.004). In addition, when using the measured compared to estimated relationships, P_max_ was significantly higher (mean difference = 25.8 ± 11.5 W, *p* = 0.003), along with a steeper slope of the moment-angular velocity data (mean difference = 5.9 ± 3.0 Nm·s·rad^−1^, *p* = 0.02). No significant differences were found in the calculated *ω*_max_ for measured compared to estimated relationships (mean difference = 0.376 ± 0.438 rad·s^−1^, *d* = 0.303, *p* = 0.398). The average fit of the linear regression relationship was similar when using the measured compared to estimated peak isometric moment (r^2^= 0.84 ± 0.08, r^2^ = 0.88 ± 0.07 for measured and estimated relationships, respectively, *p* = 0.127). Therefore, using the measured peak isometric moment to calculate the slope of the moment-angular velocity-power relationship was preferred.

#### 3.1.6 Force-power-velocity relationship

**Figure 6:**
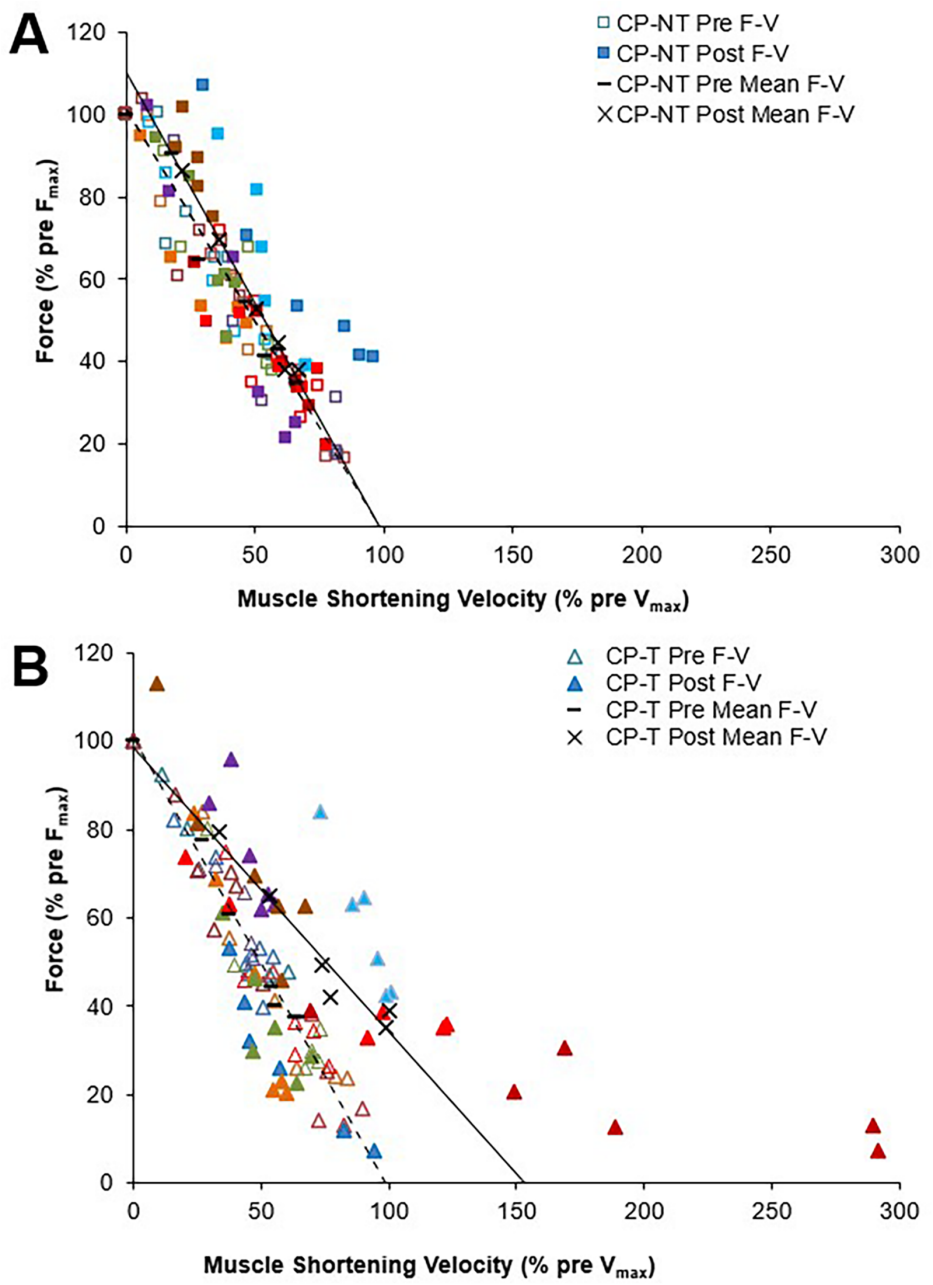
Mean force-velocity (F-V) relationships for pre (dashed line symbol) and post (straight line) training in individuals with CP not performing the training **(A)** and in individuals with CP performing the training **(B)** each participant is represented by a different color. Force and muscle shortening velocity data are presented as a percentage of pre-training maximum (pre F_max_ and pre- V_max_, respectively).

To assess differences between groups for peak muscle shortening velocity (mm/s) and muscle force (N), muscle shortening velocity was expressed relative to pre-intervention (training or no-training) values (Figure 6). V_max_ and V_opt_ were increased by 45.2 ± 76.4% following training in the CP-T group; however, this increase was not significantly different between groups (d = 0.909, *p* = 0.452). In contrast, V_max_ was unchanged (+2.9 ± 70.5%, *d* = 0.059, *p* = 1.00) following the intervention period in CP-NT. A very large effect size was also observed in V_opt_ in CP-T following training (+22.6 ± 12.4%, *d* = 0.909, *p* = 0.452), compared to CP-NT (−1.5 ± 12.4%, *d* = 0.059, *p* = 1.00). There were no differences in F_max_ between CP-T or CP-NT following the intervention (mean difference = −0.03 ± 0.1%, *d* *=* −0.117, *p* = 0.746), and the slope of the linear force-velocity relationship was similar between groups (mean difference = −0.14 ± 0.15, *d* *=* 0.342, *p* = 0.35).

**Figure 7:**
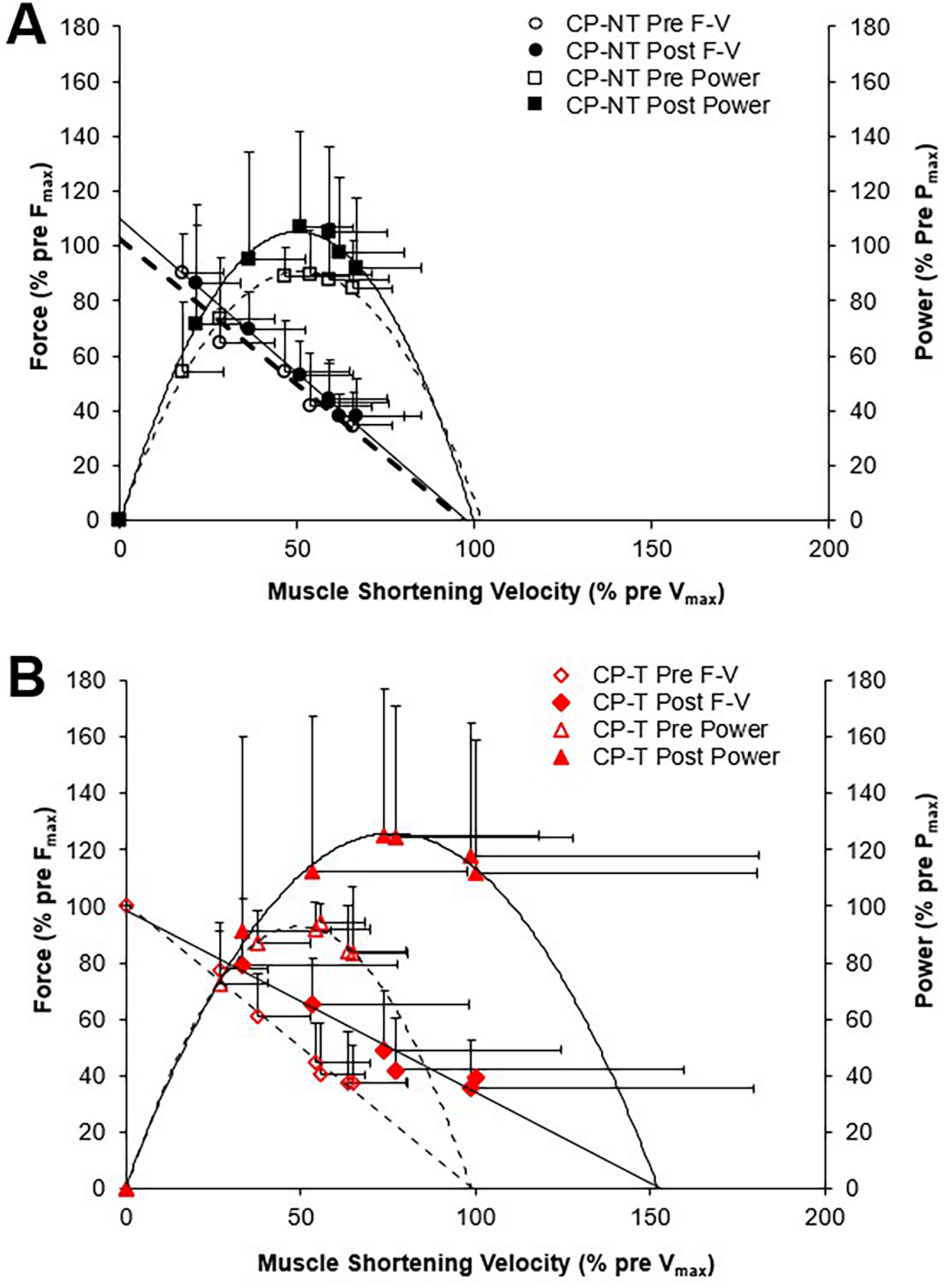
Mean force-velocity (F-V) from which mean power-velocity relationships were calculated. Power-velocity data were calculated from individual data shown in Figure 6 for the pre (dashed line, open symbol) and post (straight line, filled symbol) intervention periods. Data are mean ± SD. **(A)** CPNT (black □, ■), **(B)** CP-T (red Δ, ▲).

Force-velocity-power relationships for CP-T and CP-NT groups are shown in Figure 7. There was a very large effect size for P_max_ following the intervention period in CP-T (35.0 ± 49.1%, *d* = 1.093, *p* = 0.232), and only a small effect observed following the intervention period in CP-NT (7.8 ± 49.1%, *d* = 0.245, *p* = 1.00). Training resulted in 5 of the 8 (62.5%) CP-T participants increasing their P_max_ (mean change across 5 participants = 355.0 ± 349.9W). These increases were primarily due to increases in V_max_ in all 5 participants. In the CP-NT, 2 of the 8 participants (25%) increased their P_max_ (mean change across 2 participants = 1,179.0 ± 873.1W) following the 10-week no training period.

A three-way repeated measures ANOVA showed no three-way (group x time x isokinetic trial) interactions, no two-way (group x time, group x trial, trial x time) interactions, or main effect of group for muscle shortening velocity (*p* = 0.207), muscle force (*p* = 0.388), and muscle power (*p* = 0.239). A *post hoc* comparison for group x time indicated a moderate effect size for peak muscle shortening velocity following training in CP-T (22.3 ± 11.9%, d = 0.690, *p* = 0.484), compared to CP-NT which appeared unchanged (1.1 ± 11.9%, d = 0.035, *p* = 1.000). *post-hoc* analysis of group x time showed similar force levels pre to post intervention for CP-T (2.1 ± 3.2%, d = −0.117, *p* = 1.00) and CP-NT (5.1 ± 3.2%, d = −0.287, *p* = 1.00). *post hoc* comparison of group x time indicated a large effect size for mean peak power across the isokinetic MVCs in CP-T following training (28.3 ± 13.8%, *d* = 0.844, *p* = 0.302), and medium effect size observed in CP-NT following no-training (14.4 ± 13.8%, *d* = 0.431, *p* = 0.943).

### 3.2 Part II – comparing CP to HA

#### 3.2.1 Participant characteristics

There were no significant differences between HA and CP-T groups for age (*p* = 0.651), height (*p* = 0.908) and weight (*p* = 0.509). Total ROM was significantly lower in CP-T compared to HA (CP-T: 45.6 ± 13.1°, HA: 67.22 ± 5.91°, *p* < 0.001). In addition, significant differences in maximum dorsiflexion (CP-T: 85.6 ± 9.8°, HA: 75.4 ± 4.11°, *p* < 0.005) and plantarflexion (CP-T: 131.2 ± 8.6°, HA: 142.6 ± 4.13°, *p* < 0.001). Triceps surae moment arm was significantly longer in healthy adults (35.4 ± 3.6 mm) compared to the CP-T group (29.1 ± 5.5 mm, *p* < 0.01).

#### 3.2.2 Passive moment-length relationship

**Figure 8:**
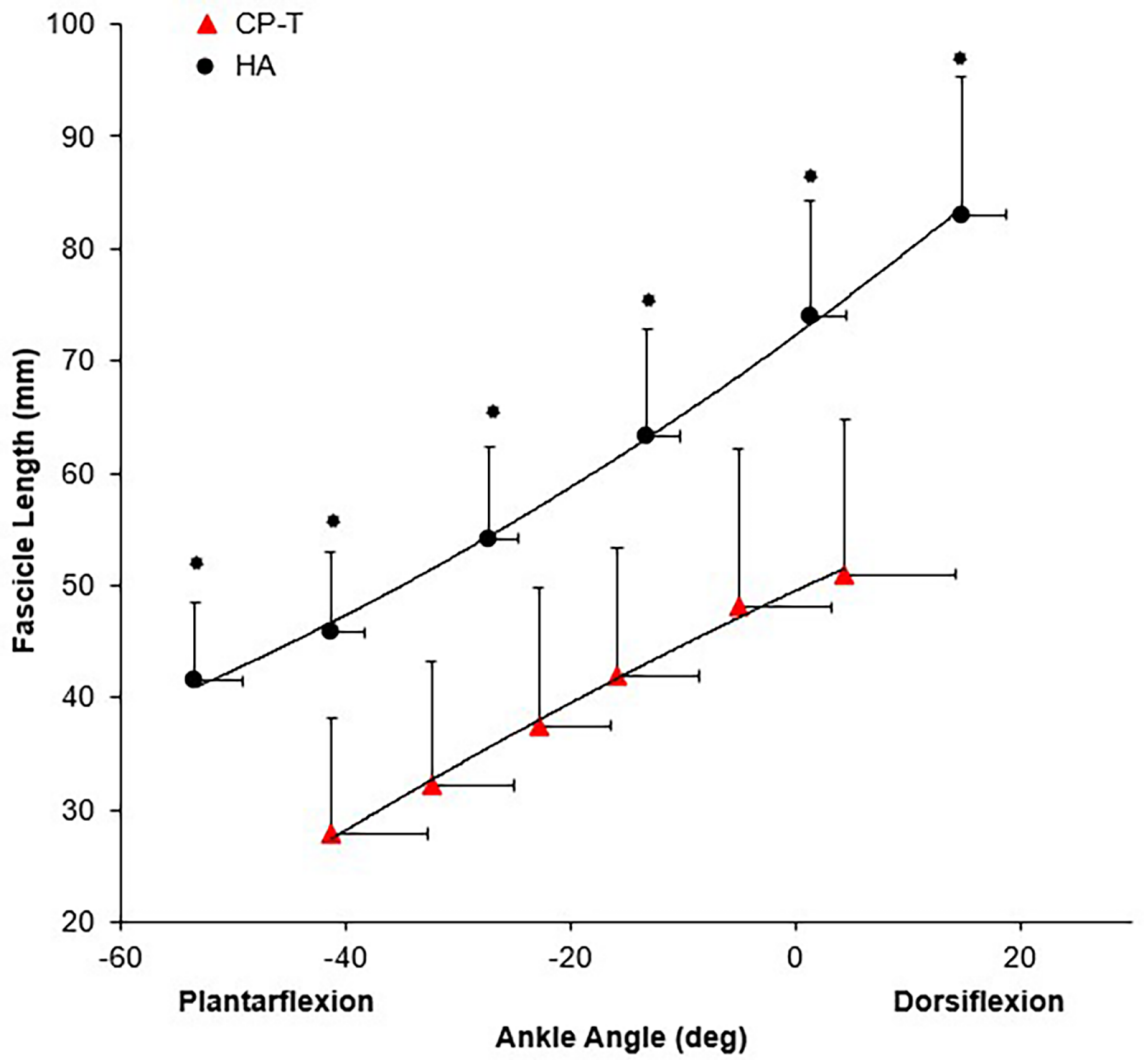
Mean resting fascicle length-angle relationship for CP-T (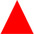) and HA (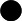) for the MG muscle during passive ankle rotation. Data are mean ± SD for CP-T (*n* = 8) and HA (*n* = 10). 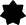 Indicates significant group x ankle angle interaction (*p* < 0.001).

Passive moment was significantly higher in CP-T across all ankle angles compared to HA (*p* < 0.05). Co-activation ratio of the TA muscle to MG was significantly higher in the CP-T group (49.6 ± 34.0%) compared to HA (18.3 ± 12.2%, *p* = 0.014). There was a significant group x ankle angle interaction for resting fascicle length (*p* < 0.001), where fascicle length was significantly longer in HA compared to CP-T across all ankle angles (Figure 8).

#### 3.2.3 Active moment-length relationships

Mean primary outcomes for HA and CP-T during isometric MVCs are found in Table 3.

**Table 3:** Mean primary outcomes during isometric MVCs for HA and CP-T.

Variable	HA	CP-T	*p*-value
Peak isometric moment (Nm)	146.81 ± 49.9	55.31 ± 23.8	<0.001*
Peak force (N)	4,237.46 ± 1,224.9	1,720.22 ± 646.7	<0.001*
Muscle thickness (mm)	18.15 ± 2.0	13.96 ± 2.1	0.001*
Pennation angle (deg)	16.21 ± 2.8	21.06 ± 6.1	0.046*
Active fascicle length (mm)	37.36 ± 6.6	29.25 ± 4.8	0.010*
Muscle fascicle Shortening (%)	48.08 ± 8.6	45.63 ± 11.9	0.652
EMG RMS LG	0.347 ± 0.201	0.111 ± 0.113	0.006*
EMG RMS TA	0.064 ± 0.046	0.043 ± 0.021	0.284

Values are mean ± SD.

* Indicates significant between group differences (*p* < 0.05).

Abbreviations: HA, healthy adults; CP-T, cerebral palsy trained group; EMG, electromyography; RMS, root mean square; LG, lateral gastrocnemius; MG, medial gastrocnemius.

**Figure 9:**
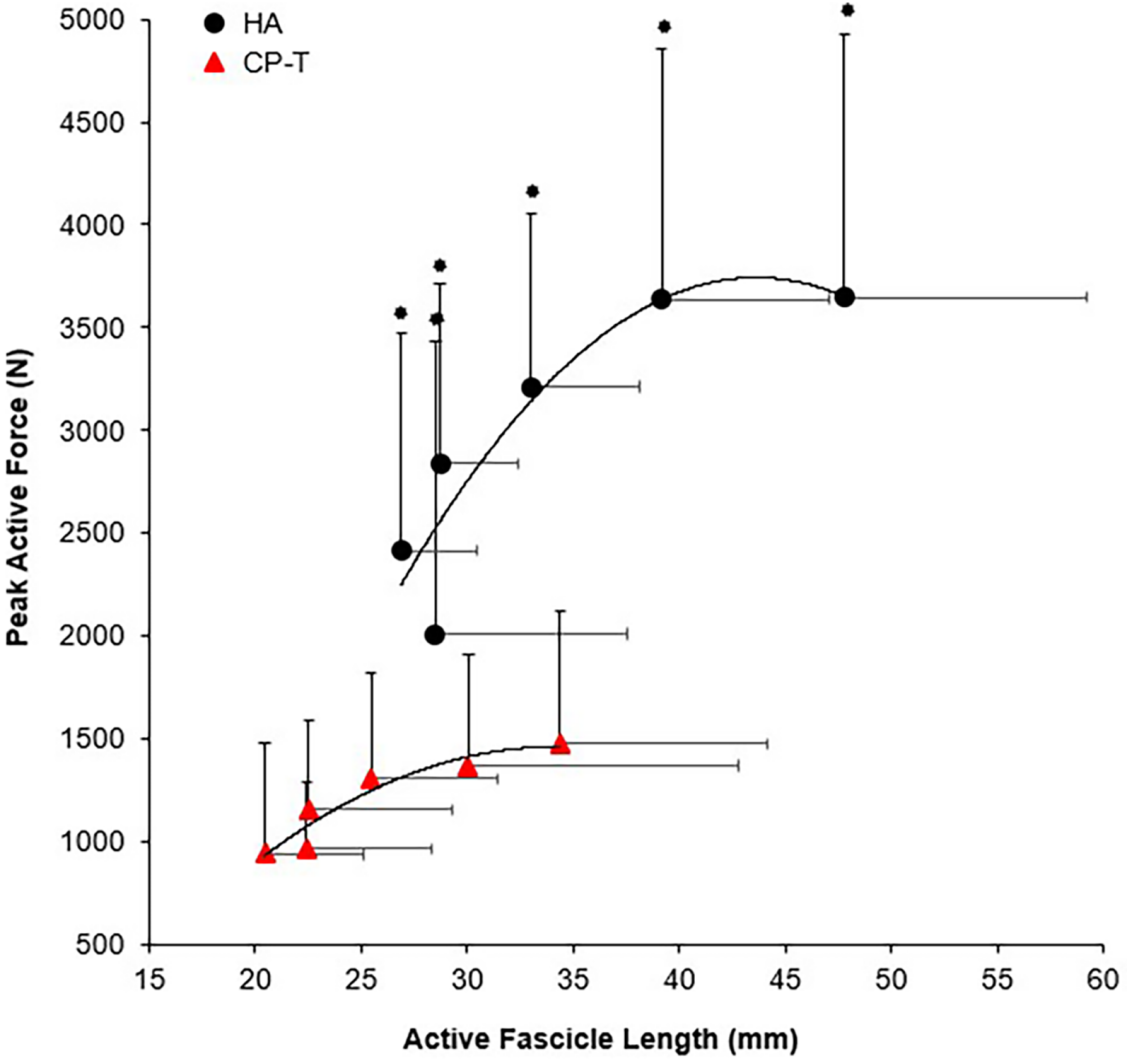
Fascicle force-length relationships of the MG muscle for CP-T (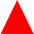) and HA (●) during isometric MVCs. Data are mean ± SD for CP-T (*n* = 8) and HA (*n* = 10). 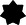 Indicates significant group x ankle angle interaction (*p* < 0.001).

Peak active force and peak isometric moment were significantly higher across all ankle angles in HA compared to CP-T (*p* < 0.001), as was active fascicle length (*p* = 0.010). Force-length relationships for CP-T and HA can be found in Figure 9.

#### 3.2.4 Moment-power-angular velocity relationships

**Figure 10:**
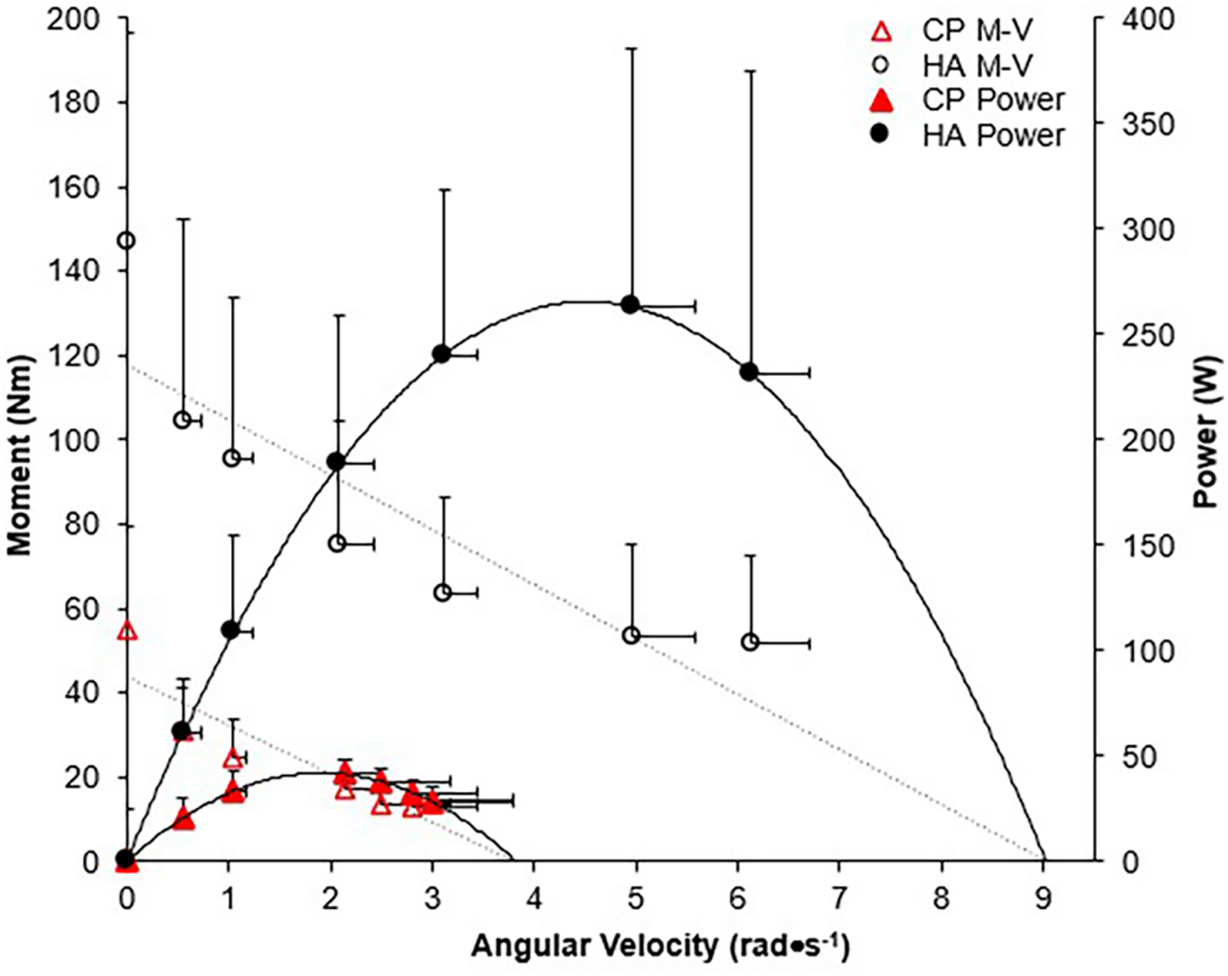
Moment-angular velocity (dotted line, open symbols) and power-angular velocity relationships (straight line, filled symbols) for CP-T (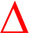, 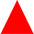) and HA (o, ●) during isokinetic MVCs. Power was calculated from the linear moment vs. angular velocity relationship, derived from mean participant data. Data are mean ± SD for CP-T (*n* = 8) and HA (*n* = 10).

There was a significant group x isokinetic trial interaction for ankle angular velocity (*p* < 0.01), moment (*p* < 0.01), and power (*p* < 0.02), indicating significantly higher outcomes across all isokinetic velocities for HA as compared to CP-T (Figure 10).

## 4 Discussion

One of the first of its kind, this study measured the active triceps surae muscle force-length and force-velocity-power relationships following 10 weeks of high-velocity sprint and jump activities in young adults with CP. These relationships highlight the compromised isometric and velocity dependent force generation capability for young adults with CP, when compared to HA without a neurological disorder. One of the key findings was that HVT over a 10-week period resulted in a significantly increased resting fascicle length in participants with CP compared to pre-training (+1.92 mm, *p* < 0.005). Although our original hypothesis was that increases in fascicle length would result in increased muscle shortening velocities and muscle power outputs, no significant changes in these metrics were found.

### 4.1 Inter-individual variations with training

Research in HA (15), older adults (41) and stroke patients (20) have shown increases in muscle mechanical power output after resistance training with a focus on speed of movement during the resistance training. The literature suggests that increased fascicle length is a training-specific response to HVT like sprinting, as demonstrated by longer MG muscle fascicle lengths in elite sprinters (66.4 ± 13.2 mm) when compared to distance runners (53.6 ± 7.2 mm) (42). For elite sprinters, fascicle lengths are expected to exceed those observed in our HA data at similar ankle angles (61.9 ± 9.4 mm). Training specific responses to fascicle length were observed in the CP-T group, but large differences still remain when compared to HA (mean difference: −18.7 ± 5.1 mm, *p* = 0.001). Only two previous studies have evaluated changes in fascicle length with HVT for people with high-functioning CP (13, 21), and muscle fascicle length changes were inconsistent with the improvements in muscle power output observed in both studies. In Moreau et al. (2013), only the rectus femoris muscle had a differential adaptation in fascicle length in response to HVT, compared to no changes observed in the vastus lateralis muscle. Although this study suggests the use of HVT, the knee angular velocities reached during training (30–120°•s^−1^), did not meet those observed in other studies during self-selected walking pace in CP (180–220°•s^−1^) (26, 43). The lack of fascicle length change in the Gillet et al. (2018) study, could be attributed to the combination of HVT with heavier resistance exercises (slower velocity), and/or attributed to the low volume (2–3) high-velocity training exercises used in the protocol.

**Figure 11:**
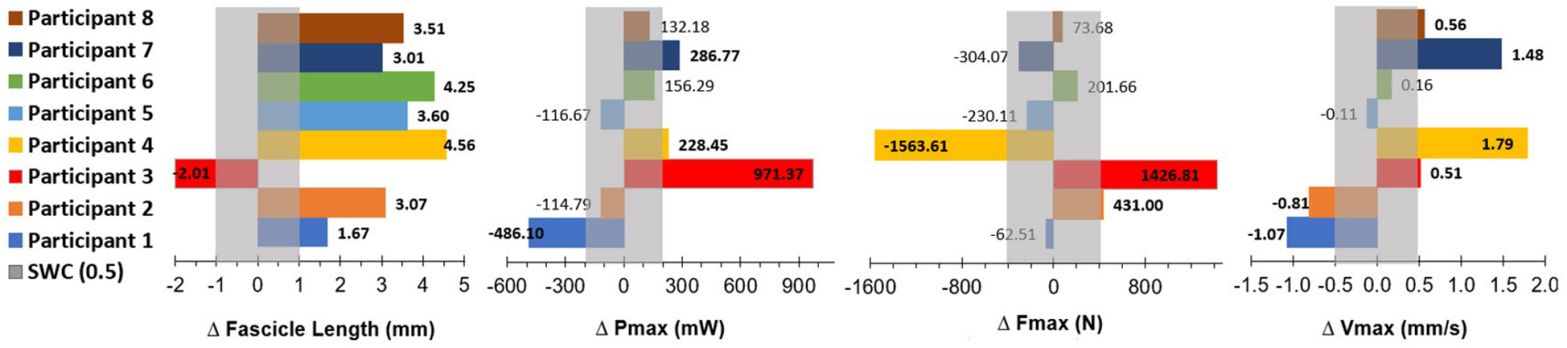
Data are pre-training values subtracted from post-training values (positive bold numbers indicate a change greater than the SWC after the intervention). SWC is indicated as the grey bar in each graph*,* and calculated as SWC = 0.5xSD. P_max_, peak power; mW, milliwatts; F_max_, peak isokinetic force; N, Newtons; V_ma_x, peak muscle shortening velocity; mm/s, millimeters per second; SWC, smallest worthwhile change (0.5*SD).

The variability observed among participants across training studies may stem from differences in training protocols, but can also be attributed to individual-specific adaptive responses to training stimuli (44, 45). Our training intervention in CP was no exception, with HVT resulting in considerable individual variations in change scores, as observed by the large range and standard deviations in values. The smallest worthwhile change (SWC) is indicated by a grey bar in Figure 11, where a level of 0.5 was used to ensure the SWC was higher than the calculated standard error of the mean (SEM= $\frac{\mathrm{SD}}{\sqrt{\mathrm{sample}\;\mathrm{size}}}$). HVT resulted in structural improvements (increases in fascicle length) in 7 of the 8 participants, and functional improvements (increases in P_max_, F_max_ and/or V_max_) in 5 of the 8 participants. 2 of the participants who had an increased fascicle length following the training period, did not demonstrate any significant functional improvements. This variation in response is not a result of adherence to the training sessions, or differences in intensity and volume for each participant, as all participants completed the same standardized HVT program with high (≥95%) adherence levels. It can be expected that a small proportion of participants will respond negatively or not at all to training, as the HERITAGE Family study by Barber et al. (2022) found the distribution of negative (low) training response scores to be approximately 4.5% across four phenotype traits (45). The results of this study were slightly higher, with approximately 12.5% (*n* = 1) of participants having a negative response to training.

### 4.2 Force-length relationships

The force producing capability of muscle is influenced by several factors, and in this study, individuals with CP achieved an F_max_ of only 36% relative to the HA group. These findings are supported by a significant reduction in triceps surae muscle MA (∼6.5 mm smaller), which would result in a decreased mechanical advantage of the triceps surae in producing effective moments during walking/running. A shorter MA would also result in a reduced muscle excursion for a given ankle joint rotation, allowing a slower muscle shortening velocity to achieve a given joint angular velocity. A slower muscle shortening velocity, while reducing maximal mechanical power output, would also reduce muscle activation needed to achieve a given muscle force. Our results revealed LG muscle activity to be significantly lower in CP-T (32% relative to HA, *p* = 0.006), along with a significantly higher plantarflexion co-activation ratio in CP-T (49.6% TA:LG) relative to the HA (18.3% TA:LG). The reduction in maximal ankle plantarflexion force may also be attributed to the increased passive force observed in the CP group, compared to HA. Other researchers have linked these differences in passive force to longer sarcomere lengths (6–8) and/or increased connective tissue and collagen deposition (6, 46).

**Figure 12:**
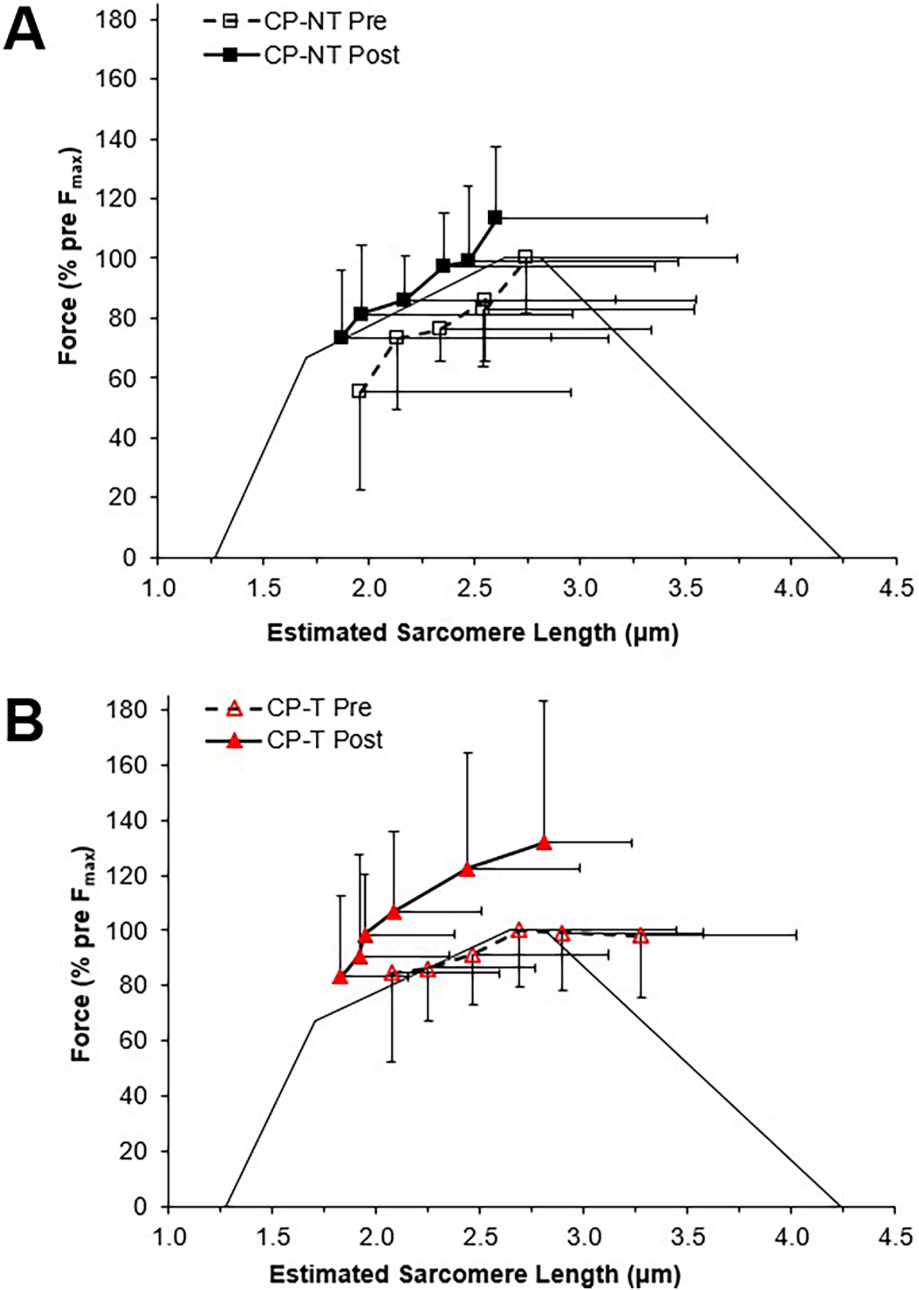
Estimated sarcomere force-length relationships for pre (dashed line, open symbol) and post (straight line, closed symbol) interventions. Data are mean ± SD. Active force is represented as a percentage of pre-intervention peak. **(A)** CP-NT (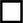, 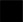). **(B)** CP-T (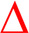, 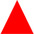).

The force-length relationship of muscle has been used in the literature as an indicator for longitudinal muscle fascicle growth (39, 47). A rightward shift in this relationship would indicate peak forces occurring at longer muscle lengths and suggest fascicle length has increased. This rightward shift was not observed in the force-length relationships for the CP-T or CP-NT groups (Figure 4), instead an upward shift in the CP-T group indicated participants were able to reach a higher percentage of their peak force at similar fascicle lengths following the training intervention. In the CP-NT group, a leftward shift resulted in peak force occurring at significantly shorter fascicle lengths following the intervention (*p* < 0.05), and aligns with data indicating a reduced resting fascicle length following the no-training period (*p* < 0.013). Considering whole muscle force-length relationships are accurately modelled as scaled sarcomeres, it's possible to relate these relationships to the sarcomere force-length relationship (48). The literature has suggested individuals with CP may have fewer sarcomeres in series for a given muscle length, and this would result in a relationship where sarcomeres operate on the descending region (longer sarcomere lengths) during contraction. This relationship was observed prior to interventions in 5 of the 8 CP participants (3 CP-T, 2 CP-NT), and not in any of the CP-T participants following training. Sarcomeres that operate on the ascending and plateau region of the relationship during contraction are considered to be at optimal length, as force capability is near maximal or maximal. This relationship was present in all (*n* = 8) CP-T participants following training, indicating an improvement in muscle force-length properties with HVT. It is also apparent that the CP-T group may be operating at shorter sarcomere lengths (on the ascending limb) during contraction, which may indicate an increase in number of sarcomeres in series (Figure 12).

### 4.3 Force-velocity-power relationships

Our data confirm previous findings that individuals with CP produce a reduced MG force at higher velocities of movement (49) resulting in lower muscle power output. In addition, the maximum isokinetic angular velocity (estimated from a linear regression analysis) was significantly lower in the CP group (166.2 ± 43.2 deg•s^−1^), compared to the HA (351.0 ± 33.6 deg•s^−1^, *p* < 0.001). The goal with HVT would be to increase the muscles capability to shorten at higher velocities, which in turn would increase walking speeds and the ability to run and jump at higher (sport-specific) velocities. It is expected that a muscle with longer muscle fascicles will generate a greater force at the same absolute shortening velocity, as the shortening speed of each sarcomere in a fascicle would be relatively slower for a given speed of whole fiber shortening (13, 16). The mechanical power producing capability of a muscle can be evaluated through the force-velocity relationship, where increases in muscle force and/or muscle shortening velocity will result in increased peak power output. Therefore, an increased muscle fascicle length should result in a rightward shift of the force-velocity relationship, as observed following the training intervention in CP-T (estimated v_max_ increased by approximately 45%), but overall, no significant changes in P_max_, v_max_ and F_max_ were observed. The variability in response between groups contributed to the lack of significant changes observed, and can be partially attributed to an increase in P_max_ in 2 CP-NT participants following the 10-week (no-training) period. One of the two CP-NT participants reported completing training for an international para track cycling competition (change in P_max_ =  + 1,796.41 W) during the no-training period, which would have included training at high power outputs. The second CP-NT participant completed the training intervention first, and any post-intervention extracurricular activity or lagging performance improvements is unclear. The use of force-length and power-velocity relationships provided meaningful insights into the implications of HVT, suggesting this protocol will at the very least slow decrements observed in CP muscle structure and function, and more importantly, highlight performance improvements for CP-T participants.

### 4.4 Limitations

In this study, we focused specifically on the potential to increase MG fascicle lengths following HVT, with the notion that increased MG fascicle lengths would imply more sarcomeres in series. The latter has the potential to amplify MG muscle power output (the product of plantar flexor force and muscle-tendon shortening velocity) as a result of higher maximal shortening velocities. Because the aim of this study was to examine the impact of HVT on MG fascicle length changes, we did not specifically quantify the impact of other factors that might influence mechanical power output of the plantar flexors. For example, we did not specifically examine changes in Achilles' tendon properties as a result of HVT, which we acknowledge may also increase plantar flexor mechanical power output because tendons can return a portion of the mechanical strain energy stored during tendon stretch (50). Mechanical power output may also be elevated following HVT due to changes in neuromuscular activation (51) and/or total muscle volume (52). Future research incorporating direct assessments of these properties would provide a more comprehensive understanding of triceps surae function following HVT.

It is not possible to assume this training intervention only consisted of high velocity shortening contractions, as the participants were instructed to control their jump landings and decelerate after their sprints to minimize the chance of injury. Lengthening contractions under load have also been found to broaden the force-length relationship or the working range of muscle length in CP (53), which we can also not discount affecting the meaningful increase in fascicle lengths following the intervention period. Fascicle length increases were used to infer that increases in serial sarcomere number (sarcomerogenesis) were observed, but sarcomeres in series and sarcomere lengths were not directly measured in this study. This inference could be more substantial in this population, as sarcomere lengths have been found to be longer in individuals with CP (6–8). Visualizing and tracking muscle fascicles using ultrasound imaging can also be quite challenging in this population, due to altered muscle pathology (increased connective tissue and collagen deposition) (6, 46). These concerns are lessened in trained populations with higher muscle quality (54). To overcome these challenges, considerable care was taken, both in placement and individual ultrasound settings of depth, focus, power and gain to optimize the imaging of, and subsequent measurement of, individual MG fascicle lengths. Exemplar images of resting MG fascicles are shown in the Supplementary Material. The measurement of muscle fascicle lengths has been shown to be reliable across a broad range of experimental conditions (55), including in individuals with CP (56–59).

We did not directly measure muscle shortening via ultrasonography during the training intervention. We make the assumption that the joint angular velocities during the running, sprinting and jumping in this training program were truly high velocity, and above the maximal plantarflexion push-off velocities found in CP during walking (180–220°·s^−1^) (43).

Using exercises that could be implemented within the daily training environment was a priority for researchers of this study to be able to make realistic inferences on high-velocity training to changes to muscle architecture, and accompanying functional and performance changes. In a recent review of the literature, Davis et al. 2020 supported the development of exercise protocols using optimal training conditions (including exercise at an appropriately high velocity) in order to increase fascicle lengths by increasing the number of serial sarcomeres in spastic CP muscle (59). Specifically, the training protocols should satisfy the following: (1) involve eccentric exercise at relatively high velocity; (2) result in stretching of the muscle fascicles and (3) momentary deactivation of the stretched muscle (59). Our training protocol emphasized high-velocity muscle shortening (concentric) contractions, to stimulate increases in muscle fascicle length. It is important to acknowledge, that eccentric contractions could not be entirely eliminated during the HVT exercises, therefore, some fascicle stretch under load likely occurred. It is possible that the training conditions used in our study may not have satisfied all of these criteria, and thus been insufficient to observe structural or functional changes in all participants. Because we did not directly measure the stretching and activation of muscle *during* the training, the precise mechanisms for increased sarcomerogenesis with relatively high velocity training remains unknown. Some participants may have benefited from an increased frequency of sessions (> 2 sessions/week), or a longer training period (>10weeks). The variability in response of each group could also be attributed to our limitations to adequately control for training or physical activity performed outside of the training and control interventions. Lastly, the smaller sample size combined with high variability observed in our study may have precluded achieving statistically significant differences, particularly for the differences in P_max_ across training and CP vs. HA groups. We have also reported effect sizes for this reason.

## 5 Conclusion

Although substantial differences between HA and CP remained after 10 weeks of HVT, our findings support the use of HVT as a viable intervention for improving muscle fascicle length, which may indicate an increase in sarcomeres in series in this population. Our study adds to the literature, how changes in fascicle length influence muscle function through force-length and force-velocity relationships of the plantarflexors in CP. Our approach prioritized exercises that are feasible in daily training environments, making the findings more applicable to the clinical management of CP. This practical application also allows practitioners the opportunity to broaden training prescriptions and target specific muscle architectural and functional changes. Additionally, our results highlight that high-functioning individuals with CP can safely perform maximal exertion exercises at high velocities, offering guidance for clinicians and strength & conditioning coaches aiming to incorporate this type of training. While promising architectural and functional adaptations to HVT were observed, future research should investigate prolonged training or the incorporation of movement-specific training and testing to further reduce the gap that exists between HA and CP. Overall, this study represents a key step toward more targeted and intensive training programs for individuals with CP, with the aim of improving both daily functioning and sport performance.

## Data Availability

The raw data supporting the conclusions of this article will be made available by the authors, without undue reservation.

## Supplementary material

Supplementary Figure S1 Exemplar ultrasound images of resting MG fascicle lengths in a participant with CP (left) and a HA participant (right). Yellow dashed line shows the measured fascicle at rest.
