# Supplementary figures and images for: Muscle fascicle length adaptations to high-velocity training in young adults with cerebral palsy
Source: Front Sports Act Living. 2025 May 13;7:1558784. doi: 10.3389/fspor.2025.1558784 (PMC12106427; doi:10.3389/fspor.2025.1558784)

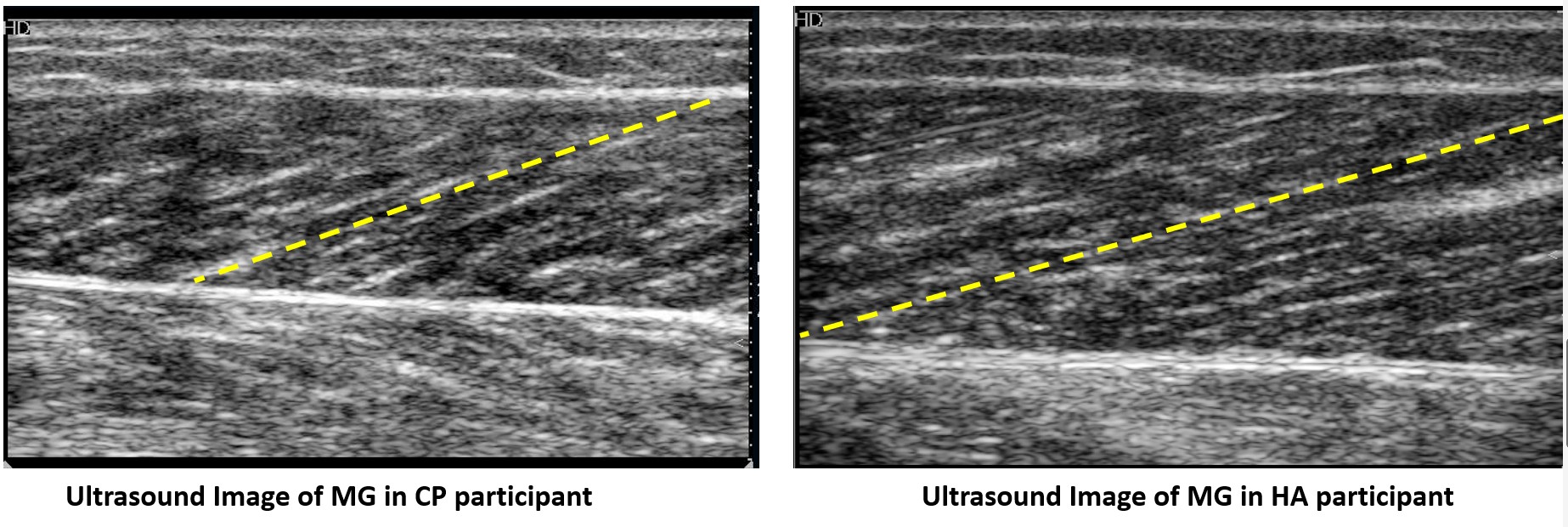

Supplement: Supplementary Figure S1 — Exemplar ultrasound images of resting MG fascicle lengths in a participant with CP (left) and a HA participant (right). Yellow dashed line shows the measured fascicle at rest. [file Image1.jpeg]
